# Combining omics tools for the characterization of the microbiota of diverse vinegars obtained by submerged culture: 16S rRNA amplicon sequencing and MALDI-TOF MS

**DOI:** 10.3389/fmicb.2022.1055010

**Published:** 2022-12-07

**Authors:** Juan J. Román-Camacho, Isidoro García-García, Inés M. Santos-Dueñas, Armin Ehrenreich, Wolfgang Liebl, Teresa García-Martínez, Juan C. Mauricio

**Affiliations:** ^1^Department of Agricultural Chemistry, Edaphology and Microbiology, Agrifood Campus of International Excellence ceiA3, University of Córdoba, Córdoba, Spain; ^2^Department of Inorganic Chemistry and Chemical Engineering, Agrifood Campus of International Excellence ceiA3, Nano Chemistry Institute (IUNAN), University of Córdoba, Córdoba, Spain; ^3^Department of Microbiology, School of Life Sciences, Technical University of Munich, Freising-Weihenstephan, Germany

**Keywords:** acetic acid bacteria, alcohol wine, craft beer, fine wine, metagenomics, protein fingerprinting

## Abstract

Vinegars elaborated in southern Spain are highly valued all over the world because of their exceptional organoleptic properties and high quality. Among the factors which influence the characteristics of the final industrial products, the composition of the microbiota responsible for the process and the raw material used as acetification substrate have a crucial role. The current state of knowledge shows that few microbial groups are usually present throughout acetification, mainly acetic acid bacteria (AAB), although other microorganisms, present in smaller proportions, may also affect the overall activity and behavior of the microbial community. In the present work, the composition of a starter microbiota propagated on and subsequently developing three acetification profiles on different raw materials, an alcohol wine medium and two other natural substrates (a craft beer and fine wine), was characterized and compared. For this purpose, two different “omics” tools were combined for the first time to study submerged vinegar production: 16S rRNA amplicon sequencing, a culture-independent technique, and matrix-assisted laser desorption/ionization-time of flight mass spectrometry (MALDI-TOF MS), a culture-dependent method. Analysis of the metagenome revealed numerous taxa from 30 different phyla and highlighted the importance of the AAB genus *Komagataeibacter*, which was much more frequent than the other taxa, and *Acetobacter*; interestingly, also archaea from the Nitrososphaeraceae family were detected by 16S rRNA amplicon sequencing. MALDI-TOF MS confirmed the presence of *Komagataeibacter* by the identification of *K. intermedius.* These tools allowed for identifying some taxonomic groups such as the bacteria genera *Cetobacterium* and *Rhodobacter*, the bacteria species *Lysinibacillus fusiformis*, and even archaea, never to date found in this medium. Definitely, the effect of the combination of these techniques has allowed first, to confirm the composition of the predominant microbiota obtained in our previous metaproteomics approaches; second, to identify the microbial community and discriminate specific species that can be cultivated under laboratory conditions; and third, to obtain new insights on the characterization of the acetification raw materials used. These first findings may contribute to improving the understanding of the microbial communities’ role in the vinegar-making industry.

## Introduction

The Andalusia region, located in southern Spain, has been, since immemorial times, a production area of famous and unique vinegars. Several factors influence the organoleptic properties and quality of these fermented products (Mas et al., 2014; Ho et al., 2017). First, the climate and soil factors of this Mediterranean region allow the growing of native raw materials, including grapes, cereals, and other fruits which are used for obtaining some high-quality acetification substrates such as wines (Maestre et al., 2008; Hidalgo et al., 2013; Mas et al., 2014; Román-Camacho et al., 2022). Second, the starter culture is composed of a complex community of microorganisms and does not represent an axenic culture of a single species (Li et al., 2015; Trček et al., 2016). Normally, species of the acetic acid bacteria (AAB) genera *Acetobacter* and *Komagataeibacter* (mainly relocated from *Gluconacetobacter*) predominate in the media because of their capabilities to perform, efficiently, the incomplete oxidation of the ethanol into acetic acid, as well as their tolerance to both substrate and product, and low pH (Mamlouk and Gullo, 2013; Wang et al., 2015; Qiu et al., 2021; He et al., 2022). *Acetobacter pasteurianus* is usually one of the predominant species at the beginning of the cycle, at a higher ethanol content, but *Komagataeibacter europaeus* is subsequently imposed during the rest of a submerged acetification. This results in final products with a high acetic acid content and other metabolites that influence the organoleptic properties (Andrés-Barrao et al., 2011; Zhu et al., 2018). Moreover by metagenomic and metaproteomic approaches, new non-abundant microbial groups never identified in vinegar to date, such as other typical AAB genera and lactic acid bacteria among others, have been found along with the best-adapted predominant organisms (Trček et al., 2016; Román-Camacho et al., 2020).

The development of the submerged culture systems allowed for industrializing vinegar production and enabled to efficiently control of operational variables of the system and obtaining, rapidly, higher acid final products due to the efficiency of mass transfer and continuous vigorous aeration (García-García et al., 2009, 2019; Gullo et al., 2014). Among working modes for industrial vinegar-making, the semi-continuous one is mostly imposed (De Ory et al., 2004; García-García et al., 2007, 2009; Qi et al., 2014). Here, each cycle is started by a loading phase that refills the reactor with fresh medium to the working volume without exceeding a preset ethanol concentration. Then, an exhausting stage occurs depleting the ethanol in the culture broth to a preset extent. Lastly, a part of the reactor is partially unloaded and the remaining volume is used as inoculum for the next cycle (Lee et al., 2017; Jiménez-Hornero et al., 2020). Operational variables may be controlled to maintain mean substrate (ethanol) and product (acetic acid) concentrations within appropriate ranges for AAB which, because of their high sensibility to these variations, are self-selected for the specific medium according to their growing features (García-García et al., 2009, 2019).

The microbiota participating in vinegar-making influences the features of the final products (Zhu et al., 2018; Wang et al., 2022). However, the identification of the microbial composition is often hindered by the special growing conditions and metabolic characteristics of these microorganisms, mainly AAB, which carry out their normal activity in a medium contained within industrial bioreactors (Fernández-Pérez et al., 2010; Trček and Barja, 2015; Andrés-Barrao et al., 2016; Trček et al., 2016). Because AAB not always can be isolated under laboratory conditions outside their natural environment, due to a viable but non-culturable (VBNC) state, the study of their richness and biodiversity is limited, probably ignoring key species within the microbiota (Mamlouk and Gullo, 2013; De Roos and De Vuyst, 2018). Some authors have used various “omics” approaches to facilitate these studies throughout the industrial acetification process without the necessity to isolate the microorganisms while providing massive and precise information about the microbial composition and behavior during the process (Andrés-Barrao et al., 2016; Trček et al., 2016; Wang et al., 2021). Recently, our previous work allowed us to characterize, by metaproteomics, the microbiota existing in a submerged acetification process employing first, a synthetic alcohol wine medium as a reference raw material (Román-Camacho et al., 2020, 2021) and then, two natural substrates (Román-Camacho et al., 2022). Liquid chromatography with tandem mass spectrometry (LC-MS/MS) analyses revelated that the AAB genus *Komagataeibacter* was dominant throughout acetification, especially the species *K. europaeus* providing protein fractions above 70% of the metaproteome.

In the present work, the use of 16S rRNA amplicon sequencing, a culture-independent technique, and matrix-assisted laser desorption/ionization-time of flight mass spectrometry (MALDI-TOF MS), a culture-dependent technique, has been implemented. From these “omics” tools, the microbiota composition developing from a mixed starter culture during the course of three acetification processes, first on a synthetic alcohol wine medium and subsequently on two other natural raw materials (craft beer and fine wine), was characterized for the first time. Following this strategy, using submerged cultivation in a semi-continuous mode as a working method, the comparison of the composition of microbial profiles obtained throughout the vinegar production might both confirm our previous results based on metaproteomics as well as provide new findings in the field of vinegar industrial production.

## Materials and methods

### Raw materials

Three raw materials were used to perform fermentations. First, an alcoholic wine medium (AW) was prepared in the laboratory following the method of Llaguno (1991) with additional peptone (0.5 g/L) and yeast extract (0.25 g/L). Then, two natural raw materials, a high-sugar craft beer (B) (Mahou-San Miguel, Córdoba, Spain) and a dry fine wine (FW) from the Montilla-Moriles region (Bodegas Alvear S.A., Montilla, Córdoba, Spain). The fermentation media were diluted with distilled water to adjust the ethanol concentration to the working conditions [10% (v/v)]. The initial acetic acid concentration was of 0.1 ± 0.1% (w/v) for AW and 0.2 ± 0.1% (w/v) for B and FW. Additional information on the nutritional composition of natural raw materials may be found in Román-Camacho et al. (2022).

### Microorganism

The first acetification (AW) was started using an inoculum consisting of a mixed culture coming from a fully active operating industrial tank (UNICO Vinagres y Salsas, S.L.L., Córdoba, Spain) making wine vinegar. A sample harvested at the final moments of the process, at the point of highest acetic acid concentration, was used as a starter culture for subsequent acetifications (B, FW). The starter culture was adapted to each raw material by a previous phase according to Román-Camacho et al. (2022).

### Operating mode

**FIGURE 1:**
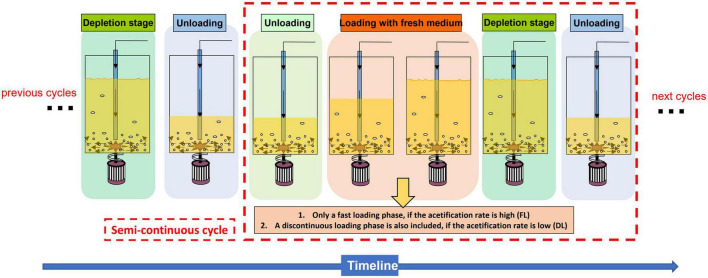
Scheme that describes the different phases that take place throughout the course of a submerged acetification process operated in a semicontinuous mode.

**FIGURE 2:**
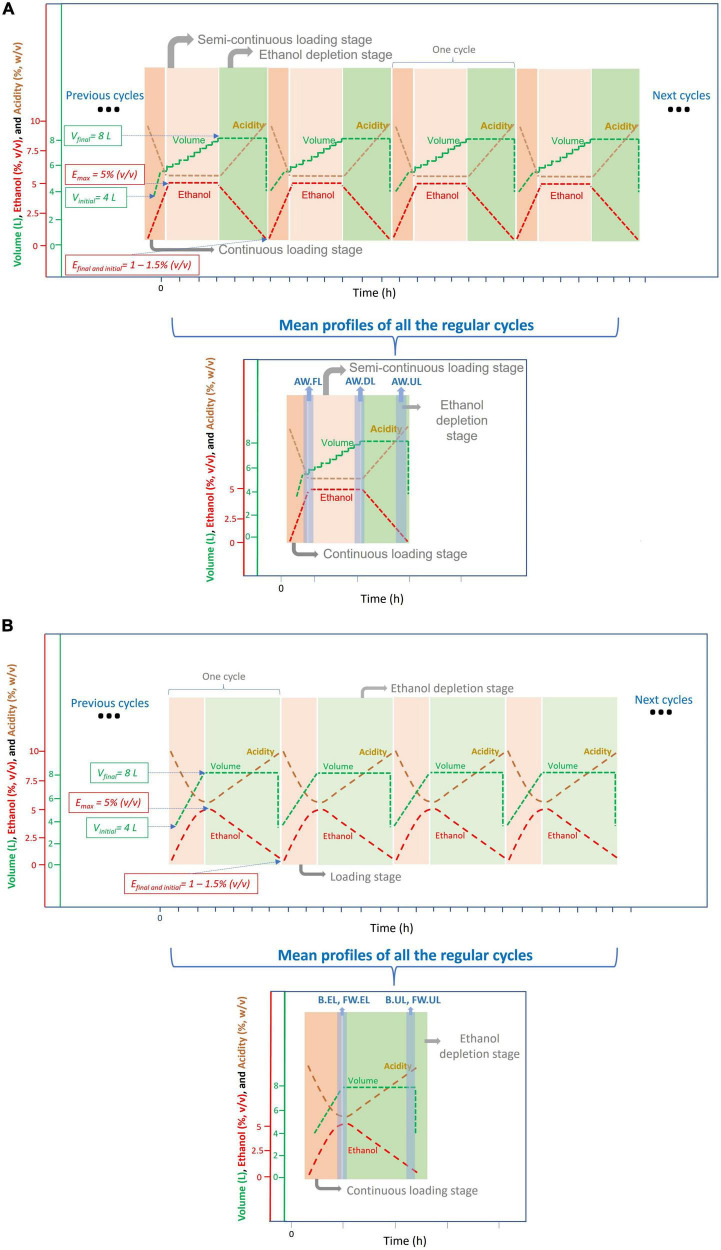
Scheme representing the operational mode used for the acetification of the different substrates; the experimental data regarding the cycle times for each acetification profile can be found in Román-Camacho et al. (2020) (for AW) and Román-Camacho et al. (2022) (for FW and B). **(A)** Submerged culture for alcohol wine vinegar (AW) production working in a semi-continuous mode. Each cycle of acetification starts by loading the tank to its working volume (8 L) without exceeding a preset ethanol concentration [5% (v/v)]. Because of a lower ethanol consumption rate in this profile, the preset ethanol concentration [5% (v/v)] is first reached (AW.FL), and therefore, an additional discontinuous loading stage (AW.DL) is necessary to reach the final working volume (8 L). When ethanol concentration is depleted to 1.0–1.5% (v/v), 50% of the reactor content (4 L) is unloaded (AW.UL). This system is maintained for the following production cycles. **(B)** Submerged culture for the beer vinegar (B) and fine wine vinegar (FW) production working in a semi-continuous mode. Each cycle of acetification starts by loading the tank to its working volume (8 L) without exceeding a preset ethanol concentration [5% (v/v)]. Because of a higher ethanol consumption rate in these profiles, the working volume is first reached (8 L) (B.EL, FW.EL) and a discontinuous loading phase is not necessary. When ethanol concentration is depleted to 1.0–1.5% (v/v), 50% of the reactor content (4 L) is unloaded (B.UL, FW.UL). This system is maintained for the following production cycles.

Acetifications were carried out in a fully automated 8 L Frings bioreactor (Heinrich Frings GmbH & Co., KG, Bonn, Germany) at a pilot scale, operating in a semi-continuous mode which works mimicking the industrial procedures as detailed in Román-Camacho et al. (2022). In order to facilitate the understanding of the operating mode, some additional figures summarizing the process are included (Figures 1, 2). A constant temperature of 31°C, a fast-loading rate of 1.3 L/h, and an air-flow rate of 7.5 L/(h L medium) were set. In the case of the beer medium, the profile was operated with a final working volume of 7 L because of excessive foaming. A detailed description of each acetification profile from a technical point of view can be found in our previous metaproteomics works (Román-Camacho et al., 2020, 2022).

### Sampling

For alcohol wine acetification, sampling was carried out at three key points: at the end of fast loading (FL), at the end of discontinuous loading (DL), and just before unloading (UL). Because of a faster ethanol consumption rate in natural raw material profiles, only two points were considered; at the end of the loading stage (EL), when the final working volume is reached, and just before unloading, at the end of the ethanol exhaustion stage (UL). A total of three or four biological replicates were taken from each point in different cycles. The starting inoculum was sampled before starting the adaptation phase, after vigorous homogenization.

### Metagenomics

#### Sample processing

Vinegar samples were harvested by directly unloading a volume of 300 mL from the acetator for each biological replicate at the sampling times aforementioned (see Section “Sampling”), dividing it into six fractions of 50 ml each, which were cooled on ice in centrifuge tubes. Cells were obtained by centrifugation and washed twice with cold sterile distilled water; the resulting cell pellets were stored at –80°C until the metagenomic procedures.

#### Genomic deoxyribonucleic acid extraction, purification, and quantification

Genomic DNA (gDNA) from all the samples was extracted by using a quick genomic bacterial DNA extraction kit (Bio Knowledge Lab, S.L., Córdoba, Spain) following the instructions provided by the manufacturer. The purity and quantity of the gDNA were determined by NanoDrop ND 1000 spectrophotometer (Thermo Fisher Scientific, MA, USA).

#### 16S rRNA sequencing

The V3-V4 region from 16S rRNA genes was amplified using the specific set of primers 341F (5′-CCTACGGGNGG CWGCAG-3′)-806R (5′-GGACTACHVGGGTWTCTAAT-3′) with barcodes (Cole et al., 2009; Caporaso et al., 2010). PCR reactions for amplicon generation were carried out with Phusion^®^ High-Fidelity PCR Master Mix (New England Biolabs, MA, USA). PCR products were mixed with the same volume of 1 × loading buffer with SYBR green and a subsequent 2% agarose gel electrophoresis was run for the detection. Samples with a bright major band at 470 bp were selected for further experiments. PCR mixed products, obtained at equal density ratios, were purified with Qiagen Gel Extraction Kit (Qiagen, Germany). The libraries were generated with NEBNext^®^ Ultra TM DNA Library Prep Kit for Illumina using the Illumina set of adapters F (5′-TCGTCGG CAGCGTCAGATGTGTATAAGAGACAG-3′)-R (5′-GTCTCG TGGGCTCGGAGATGTGTATAAGAGACAG-3′) and then, were quantified via Qubit and Q-PCR. The amplicon was sequenced on Illumina paired-end platform to generate 250 bp paired-end raw reads.

#### Raw data analysis

Raw data were firstly filtered using QIIME2 v2020.8^1^ (Bolyen et al., 2019). Reads (fastq) obtained after Illumina amplicon sequencing were denoising using the DADA2 package^2^ by conducting three steps: (1) trimming and truncating low-quality regions, (2) dereplicating the reads, and (3) chimera filtering (Callahan et al., 2016). After denoising, forward and reverse reads were merged into one sequence (fasta), dereplicated, and assigned to an ID, considering them as amplicon sequence variants (ASVs). The number of reads found at each filtering level is given in Supplementary Table 1. ASVs were grouped in operational taxonomic units (OTUs) by using the *de novo* clustering method from vsearch (Rognes et al., 2016). Clustering was carried out at 97% identity to create 97% OTUs.

The rest of the analyses were performed by using QIIME2 v2020.8. For biodiversity analysis, the core-metrics phylogenetic method, which supports computing alpha and beta diversity metrics, was used. Alpha diversity, defined as the diversity within the samples, was quantified by estimating and comparing different diversity indexes such as observed features, faith phylogenetic diversity, Shannon, and Simpson indexes. The diversity alpha rarefaction tool was applied to randomly select a different number of sequences and analyze detected OTUs at each fraction to form a rarefaction curve. Beta diversity, defined as compositional heterogeneity among samples, was represented by a Principal Coordinates Analysis (PCoA) employing the UniFrac algorithm. For taxonomic analysis, OTUs were classified by taxon using the 16S rRNA V3-V4 region SILVA database with vsearch by assigning a taxon to each OTU and generating bar plots and heatmaps with hierarchical clustering through specific QIIME2 plugins (Quast et al., 2013).

**FIGURE 3:**
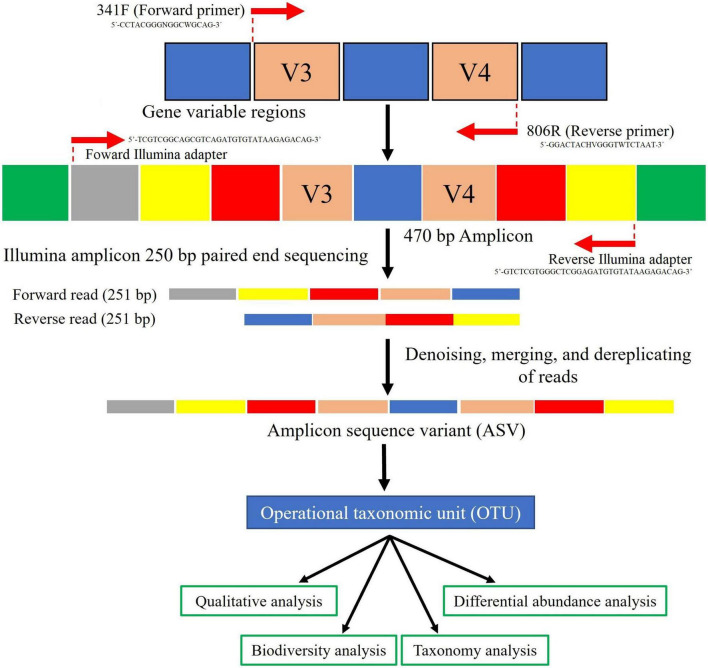
Flow diagram representing the strategy employed for 16S rRNA amplicon sequencing and raw data processing. Reads were denoising by the DADA2 package (https://github.com/benjjneb/dada2) and the rest of the procedures using QIIME2 v2020.8 (https://qiime2.org/).

Analysis of composition microbiomes (ANCOM) was applied as a statistical tool to identify differentially abundant features across sample groups at specific taxonomic levels (Mandal et al., 2015). The test calculates the number of ratios significantly different between ASV pairs [False Discovery Rate (FDR) with a *p*-value < 0.05]. The results are represented in a volcano plot, which relates the ANCOM *W* statistic to the center-log-ratio (CLR) for the groups, *W* being the number of ANCOM null hypotheses rejected for each taxon, indicating significant differences between ratios of a taxon’s relative abundance to those of *W* for other taxa. The complete strategy for 16S rRNA amplicon sequencing is summarized in Figure 3.

### Characterization of isolates by matrix-assisted laser desorption/ionization-time of flight mass spectrometry

#### Growing conditions

For each vinegar sample harvested from the reactor, a volume of 50 ml was centrifuged and washed as in Section “Sample processing”. 1 ml of cell culture was used to perform serial dilutions in distilled sterile water and subsequently, 100 μl from each dilution were cultured in an optimized GYC medium (50 g/L glucose, 20 g/L agar, 10 g/L yeast extract, 3 g/L calcium carbonate, and 0.6% of 1:1 pure ethanol and glacial acetic acid). This medium was selected after numerous assays using different media in order to obtain a high growth efficiency (Gullo et al., 2006; De Vero and Giudici, 2013). Plates were incubated at 30 °C for 6 or 7 days. Isolates were selected attending to the phenotypical features of the grown colonies including mainly morphology, aspect, color, and size. Among three and five colonies from each phenotypic group were randomly selected to try to cover the totally different microorganisms present. Then, the isolates were both grown in a fresh tube medium and incubated again under the same conditions to obtain higher biomass before identification.

#### Identification of the isolates

A qualitative analysis of the isolates was performed through MALDI-TOF MS. 5–10 mg of fresh mass from each isolate were placed in 300 μl Milli-Q water and 900 μl ethanol and then vortexed until homogenization. Samples were centrifuged at 14,500 × *g* for 2 min, pellets were dried at room temperature (RT), and subjected to 50 μl of 70% formic acid and 50 μl of acetonitrile. After a second centrifugation, proteins were obtained in the supernatant, and 1 μl was dried in a MALDI plate subsequently coated with 1 μl of HCCA matrix (α-cyano-4-hydroxycinnamic acid) prepared in a mixture of 50% acetonitrile and 2.5% trifluoroacetic acid. Samples were again dried at RT.

Dried samples were analyzed with MALDI-TOF/TOF “ULTRAFLEXTREME” (Bruker Daltonics, Bremen, Germany) equipment. Obtained spectra were treated with MALDI Biotyper Compass (MBT Compass; Bruker, MA, United States) software, calibrating the spectra before searching and matching, automatizing the measures and obtaining the identifications. Spectra were compared with reference profiles from the MBT Compass Library (Bruker) and finally, score values ≥ 1.70 were considered.

## Results

### Qualitative analysis

**FIGURE 4:**
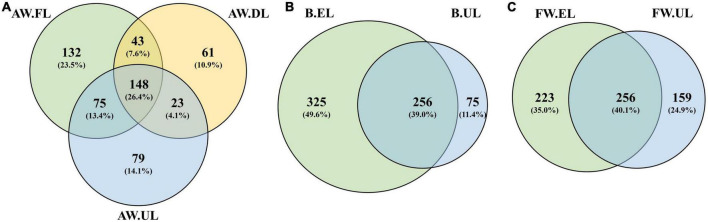
Venn diagrams representing the number of valid unique OTUs present within samples for each acetification profile. **(A)** Alcohol wine vinegar; **(B)** beer vinegar; **(C)** fine wine vinegar. AW, alcohol wine; B, beer; FW, fine wine; EL, end of loading phase (FL, fast loading; DL, discontinuous loading); UL, just before unloading.

A total of 12,443 unique ASVs were detected after performing strict quality control. After clustering and chimera filtering, a total of 6,187 unique OTUs were found at 97% identity in, at least, one out of a total of 26 samples. The distribution of the unique OTUs within samples was analyzed through Venn diagrams to obtain an overview of the qualitative metagenome for each acetification profile, see Figure 4.

For alcohol wine vinegar (AW, Figure 4A), a total of 561 OTUs were found throughout the acetification. Of them, 132 (23.5%), 61 (10.9%), and 79 (14.1%) were specific to AW.FL, AW.DL, and AW.UL phases, respectively. The highest amount of OTUs was located in the central common area for the three time points of sampling (148, 26.4%). For beer vinegar (B, Figure 4B), a total of 656 OTUs were found containing 256 (39.0%) common ones at the end of loading as well as just before unloading. In this case, the highest fraction of sampling point specific OTUs corresponded to B.EL with 325 (49.6%) contrasting with a much lower number for B.UL with only 75 (11.4%). For fine wine vinegar (FW, Figure 4C), 638 OTUs were detected in total, with 256 (40.1%) OTUs at both sampling time points, while 223 (35.0%) and 159 (24.9%) were specific to FW.EL and FW.UL, respectively, in this matrix. In general, an important fraction of OTUs was common throughout each acetification although, regarding sampling times, OTUs were more abundant at the end of loading periods (EL), especially in beer vinegar. Fine wine vinegar exhibited a higher proportion of OTUs at the final moments of the acetification (UL), amounting to around 25%, while the other profiles were characterized by a lower OTU diversity at this time point, between 10 and 15%, see Figure 4. It is also worth noting that analysis of the inoculum samples revealed a total of 919 unique OTUs, a higher number than in any sample throughout the acetification processes.

### Biodiversity analysis

**FIGURE 5:**
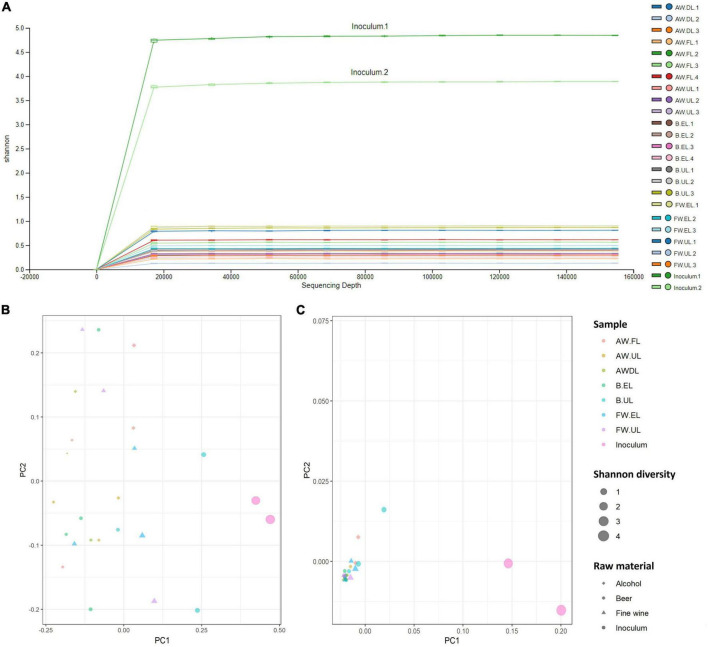
**(A)** Alpha rarefaction plot that shows the Shannon rarefaction curve as a biodiversity quantitative index. The box plots represent the distribution of the Shannon metric for each group of samples at each even sampling depth. The lower and upper whiskers of the box are the 9th and 91st percentiles, respectively, while lower and upper extents are the 25th and 75th percentiles; the horizontal bar through the middle is the median value. The line chart connects the medians of the metric distribution across sampling depths. **(B,C)** Principal Coordinates Analysis (PCoA) by using the unweighted **(B)** and weighted **(C)** UniFrac algorithm. Each sample is represented as a dot in a bi-dimensional matrix and the distance between dots indicates the similarity between samples (closer together more similarity). The color, size, and shape of dots represent sampling time, Shannon diversity, and raw material, respectively. AW, alcohol wine; B, beer; FW, fine wine; EL, end of loading phase (FL, fast loading; DL, discontinuous loading); UL, just before unloading.

The study of the biodiversity degree, provided by the amount of detected OTUs and how they are distributed both within samples and between them, is described in the following. Alpha diversity within the samples was determined through quantification by different diversity indexes including observed features, Faith phylogenetic diversity, Shannon, and Simpson indexes, see Table 1. Inoculum samples provided the highest biodiversity values in all indexes, far above acetification matrices. For alcohol vinegar (AW), the diversity pattern was similar to that shown for the OTU distribution (see Figure 4), higher at the beginning of the process (AW.FL) with a subsequent strong decrease (AW.DL) before finally increasing again but without reaching initial levels (AW.UL). For fine wine vinegar (FW), diversity was slightly higher at the end of loading period (FW.EL) compared to the sampling point just before unloading (FW.UL) while for beer vinegar (B), diversity was much higher at the final moments of the process (B.UL). These results are represented in an alpha rarefaction plot generated by randomly selecting a different number of sequences and analyzing detected OTUs at each fraction to form a rarefaction curve. The Shannon index was selected as the quantitative measure to represent the biodiversity of each sample, individually, thus connecting the median values of the metric distribution across the sampling depths, see Figure 5A.

**TABLE 1:** Alpha diversity indexes for quantification of the biodiversity degree within the vinegar samples.

Sample	Observed feat.	Faith ph.	Shannon	Simpson
Inoculum	2417 ± 784	159.3 ± 8.0	3.40 ± 1.30	0.434 ± 0.154
AW.FL	332 ± 94	49.8 ± 14.5	0.43 ± 0.16	0.067 ± 0.026
AW.DL	198 ± 56	33.6 ± 3.3	0.27 ± 0.10	0.042 ± 0.016
AW.UL	294 ± 52	42.5 ± 7.7	0.36 ± 0.04	0.054 ± 0.006
B.EL	258 ± 30	41.6 ± 4.2	0.34 ± 0.06	0.052 ± 0.009
B.UL	720 ± 197	72.9 ± 15.6	0.73 ± 0.20	0.105 ± 0.029
FW.EL	450 ± 138	52.6 ± 9.0	0.61 ± 0.19	0.104 ± 0.025
FW.UL	359 ± 158	51.9 ± 7.7	0.50 ± 0.21	0.079 ± 0.029

AW, alcohol wine; B, beer; FW, fine wine; EL, end of loading phase (FL, fast loading; DL, discontinuous loading); UL, just before unloading.

Beta diversity analyses were carried out by selecting the UniFrac algorithm both at a qualitative (unweighted) and quantitative (weighted) level and represented by a Principal Coordinates Analysis (PCoA), see Figures 5B,C. These results show a bi-dimensional matrix in which each sample is a point and the distance between them indicates the similarity between samples (closer together more similarity). The size of the points represents the diversity of Shannon ranging from level 1 to 4. The unweighted matrix (Figure 5B) showed no aggrupation of the samples which were widely distributed throughout the plot while the weighted matrix (Figure 5C) showed a very close aggrupation of most samples from each acetification profile at the bottom left although samples from the starting inoculum were particularly diverse and separated from the rest of the samples. These results might indicate that the OTUs’ frequency plays a bigger role in the samples than the OTUs’ presence/absence. From a quantitative point of view, the raw material used for making vinegar did not cause significant variations in the diversity of samples from the three acetification processes, as can be observed in their closed aggrupation.

### Taxonomy analysis

**FIGURE 6:**
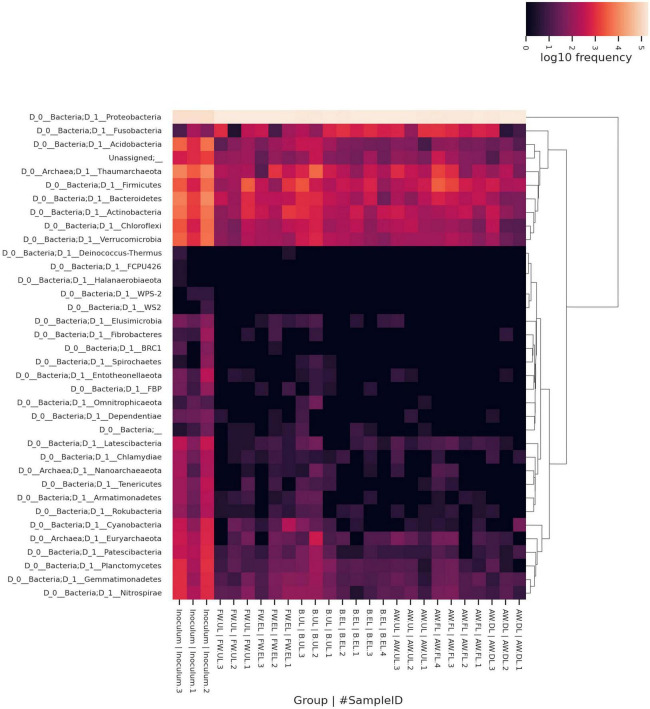
Heatmap clustering the metagenome taxonomy based on OTUs that comprise each taxon represented up to the phylum level as a maximum specificity. Taxa were grouped by hierarchical clustering for all samples according to their frequency (log10). AW, alcohol wine; B, beer; FW, fine wine; EL, end of loading phase (FL, fast loading; DL, discontinuous loading); UL, just before unloading.

The assignment of a taxon to each OTU found in the vinegar samples enabled the identification of the taxon composition within the metagenome. The total OTUs were first clustered in a heatmap obtained through the QIIME2 heatmap plugin and then also depicted in a bar plot obtained through the QIIME2 taxa bar plot plugin. In the heatmap, the taxa were grouped by hierarchical clustering and represented at the phylum level for each sample including their frequency (log10), see Figure 6. The results indicated that Proteobacteria (96.41%) was the most frequent phylum throughout the three acetification profiles (AW, B, FW), far above the rest of the phyla and being less abundant in the inoculum samples. For this reason, this group built a separate cluster from the rest of the taxonomic groups. None of the remaining phyla contributed a mean frequency ≥ 1%. Interestingly, the second most frequent phylum was Thaumarchaeota (0.69%) belonging to the archaea; with less mean frequency, another two archaeal phyla were found in this study, Euryarchaeota (0.04%) and Nanoarchaeaeota (0.01%). Some of the following most frequent taxa which conformed to a closer cluster were the bacterial phyla Bacteroidetes (0.54%), Firmicutes (syn. Bacillota) (0.51%), Actinobacteria (0.42%), Verrucomicrobia (0.27%), Acidobacteria (0.24%), Chloroflexi (0.24%) and Fusobacteria (0.22%). In general lines for these taxonomic groups, inoculum samples showed higher frequencies than any of the acetification process samples, following the results obtained in the biodiversity analysis. It is also worth mentioning that around 30 different phyla were identified by 16S rRNA gene amplicon sequencing, see Figure 6.

**FIGURE 7:**
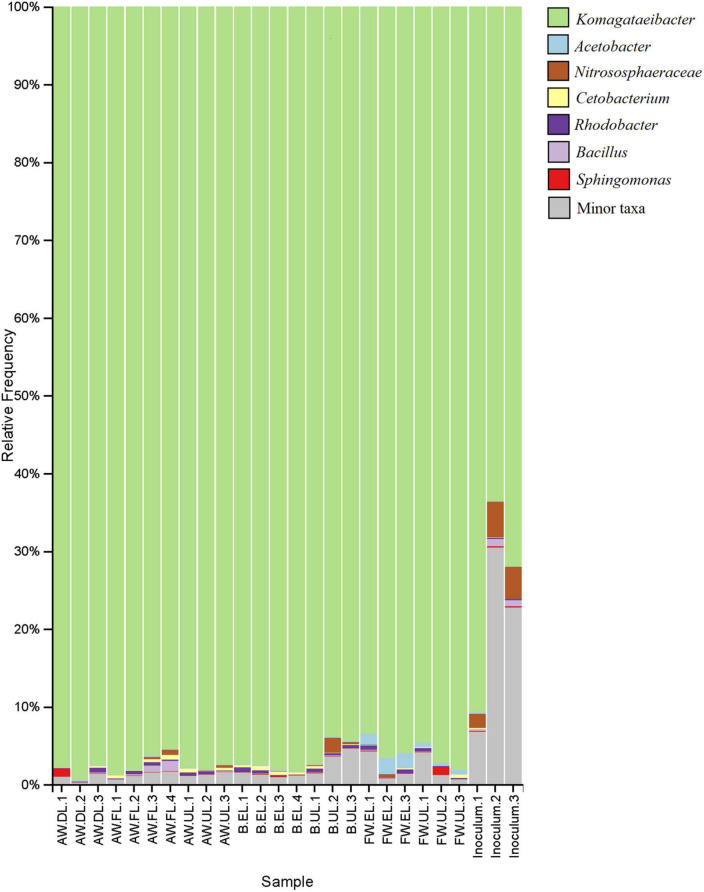
Taxonomy bar plot showing the assignment of the total OTUs to the main taxa of the vinegar metagenome. The relative frequency (%) of OTUs provided by the taxa to each sample is represented up to the genus level as a maximum specificity. The list of taxa assigned to each one of the total OTUs, including the main taxa and those conforming to a minor fraction (see gray color) can be found in Supplementary Table 2. AW, alcohol wine; B, beer; FW, fine wine; EL, end of loading phase (FL, fast loading; DL, discontinuous loading); UL, just before unloading.

The bar plot shows the relative frequency (%) that the main taxonomic groups contribute to each sample, see Figure 7. To complement the results of the heatmap and hierarchical clustering analysis, the taxa were also identified at the genus level. The acetic acid bacteria genera *Acetobacter* and *Komagataeibacter* were the main representatives of Proteobacteria. In general, *Komagataeibacter* was the most frequent taxon of the vinegar metagenomes, amounting to around 90% of the total OTUs. In the samples of the starter inoculum, the biodiversity was higher, but still *Komagataeibacter* contributed around 60–90% of the OTUs, while in the acetification samples (AW, B, FW) the predominance of the genus increased, irrespective of the raw material and the sampling time, to about 90–95%. *Acetobacter* was one of the most frequent taxa of the less abundant microbiota, especially in FW vinegar, accounting for up to 2% of the total OTUs in the FW.EL samples as well as being present in the rest of the samples. Moreover, a high difference in the number of total OTUs assigned to *Komagataeibacter* (431) and *Acetobacter* (3) might result in a significant difference in the diversity of species within the two AAB genera. Apart from these two genera, no other taxa from the acetic acid bacteria group were found among the main ones although a few other Acetobacteraceae members (e.g., *Acidiphilium, Craurococcus*, *Gluconacetobacter*, *Gluconobacter*, *Roseomonas*) were identified at very low frequency among the sequence reads (see Supplementary Table 2). The archaeal group Thaumarchaeota was found to occur as unique OTUs mainly assigned to the Nitrososphaeraceae family accounting for about 2–5% of the total metagenome in inoculum samples but were also widely found in the acetification samples. The presence of archaea in vinegar is little known and their role in the microbial community may be an object of future study. Regarding the rest of the bacterial groups of the non-abundant metagenome, the OTUs were mainly assigned to *Cetobacterium* and *Rhodobacter*, present in most of the vinegar samples, as well as *Bacillus* and *Sphingomonas*, mostly contained in specific samples of inoculum, AW, and FW. Of the microbial phyla, Proteobacteria (including the genera *Komagataeibacter, Acetobacter, Rhodobacter, Sphingomonas*) and Fusobacteria (*Cetobacterium*) provided the most frequent genera, although many other microbial groups were identified as contributing but with merely very low fractions of the total OTUs. The list of the total OTUs with the taxon assigned to each of them, including those conforming to a minor fraction of taxa (see gray color in Figure 7) is available in Supplementary Table 2.

### Differential abundance analysis

**FIGURE 8:**
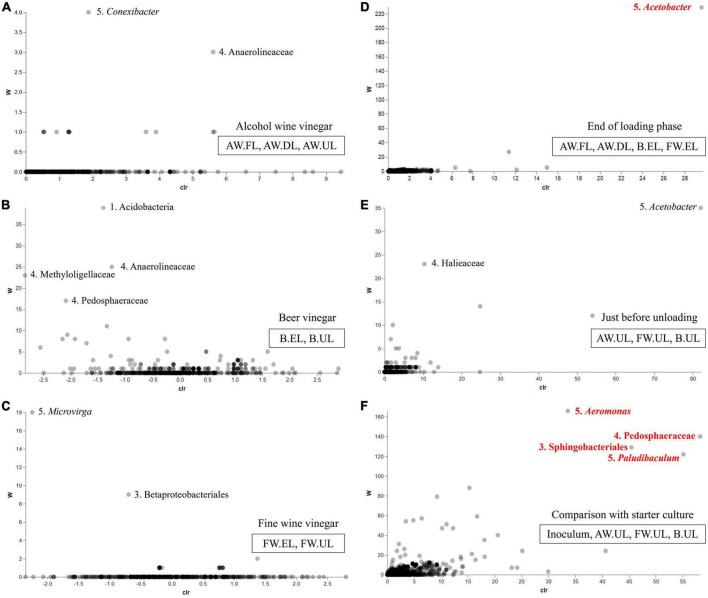
Volcano plot which represents abundance differences of vinegar metagenome through the relationship of the ANCOM *W* statistic to the center-log-ratio (CLR) for the sample groups: **(A)** alcohol wine vinegar (AW.FL, AW.DL, AW.UL); **(B)** beer vinegar (B.EL, B.UL); **(C)** fine wine vinegar (FW.EL, FW.UL); **(D)** end of the loading phase (AW.FL, AW.DL, B.EL, FW.EL); **(E)** just before unloading (AW.UL, B.UL, FW.UL); **(F)** comparison with the starter culture (inoculum, AW.UL, B.UL, FW.UL). *W* statistic is the number of ANCOM null hypotheses rejected for each taxon, indicating significant differences between ratios of a taxon’s relative abundance to those of *W* for other taxa (FDR with a *p*-value < 0.05). Taxa are displayed as dots distributed along the matrix and numbers next to highlighted taxa indicate the taxonomical category (1. Phylum, 2. Class, 3. Orden, 4. Family, 5. Genus). Those taxa with significant abundance differences are displayed in red color.

The abundance differences between the three acetification profiles and sampling times within each one of them were studied through ANCOM statistics. Figure 8 represents volcano plots with a total of six matrices (A-F) showing these differences according to the distribution of the taxa (displayed as dots) throughout each matrix. The abundance of the taxa is measured through the relationship between the ANCOM *W* statistic to the center-log-ratio (CLR). Figures 8A–C shows the alcohol wine (AW), beer (B), and fine wine (FW) acetification profiles, respectively. The AW profile (Figure 8A) and FW profile (Figure 8C) barely revealed abundance differences, although both showed a couple of taxa with slightly higher *W* values than the rest [AW: *Conexibacter* (*W* = 4), Anaerolineaceae (*W* = 3); FW: *Microvirga* (*W* = 18), Betaproteobacteriales (*W* = 9)]. The B profile (Figure 8B) exhibited more abundance differences, as reflected in a more pronounced distribution of taxa in the matrix, highlighting some taxa such as the phylum Acidobacteria (*W* = 39) and the family Anaerolineaceae (*W* = 25) among others. The end of loading (Figure 8D) and just prior to unloading (Figure 8E) samples showed low relative abundance differences among the taxa, however, the genus *Acetobacter* stood out (located at the top right of both matrices) with much higher *W* values than the rest of the taxa in its matrix, particularly among the end of the loading samples (EL), reaching a statistically significant level (*W* = 230). Analysis of the relationship between the highly different samples from starter culture and final products revealed numerous taxa with significant abundance differences highlighting the genus *Aeromonas* (*W* = 166) and the family Pedosphaeraceae (*W* = 140) among others (Figure 8F).

### Identification of isolates by protein fingerprinting using matrix-assisted laser desorption/ionization time of flight mass spectrometry

A total of 31 isolates were selected for identification after a morphological study of their colonies. Of them, 21 isolates were identified with high (2.00–3.00) or medium (1.99–1.70) confidence; 12 were from alcohol wine vinegar, 6 from beer vinegar, and 3 from fine wine vinegar. No acetic acid bacteria could be isolated on the solid medium from AW vinegar and the 12 isolates belonged to the gram-positive bacteria, *Lysinibacillus fusiformis*. A total of 7 isolates of acetic acid bacteria were obtained from samples at different phases from the acetification of natural media (B, FW), all of them belonging to the species *Komagataeibacter intermedius*. Another two strains of the bacteria species *L. fusiformis* were isolated from the FW vinegar samples. The complete list of species identified among the 31 isolates can be found in Table 2.

**TABLE 2:** List of selected vinegar isolates for identification by MALDI-TOF MS analysis.

Isolated	Species	Appearance	Sampling time	Medium	Score
1	*L. fusiformis*	Other bacteria	AW.FL	GYC	**2.11**
2	*L. fusiformis*	Other bacteria	AW.FL	GYC	**1.79**
3	*L. fusiformis*	Other bacteria	AW.FL	GYC	**2.13**
4	*L. fusiformis*	Other bacteria	AW.FL	GYC	**1.78**
5	*L. fusiformis*	Other bacteria	AW.FL	GYC	**2.08**
6	*L. fusiformis*	Other bacteria	AW.DL	GYC	**2.19**
7	*L. fusiformis*	Other bacteria	AW.DL	GYC	**2.15**
8	*L. fusiformis*	Other bacteria	AW.DL	GYC	**2.11**
9	No identified	Other bacteria	AW.DL	GYC	**1.59**
10	*L. fusiformis*	Other bacteria	AW.UL	GYC	**2.14**
11	*L. fusiformis*	Other bacteria	AW.UL	YPD	**2.28**
12	*L. fusiformis*	Other bacteria	AW.UL	YPD	**2.29**
13	*L. fusiformis*	Other bacteria	AW.UL	GYC	**2.23**
14	No identified	Other bacteria	AW.UL	GYC	**1.62**
15	No identified	Other bacteria	AW.UL	GYC	**1.43**
16	*K. intermedius*	AAB	B.EL	GYC	**1.80**
17	*K. intermedius*	AAB	B.EL	GYC	**1.70**
18	No identified	AAB	B.EL	GYC	**1.34**
19	No identified	AAB	B.EL	GYC	**1.54**
20	No identified	AAB	B.EL	GYC	**1.54**
21	*K. intermedius*	AAB	B.EL	GYC	**1.71**
22	*K. intermedius*	AAB	B.EL	GYC	**1.81**
23	No identified	AAB	B.UL	GYC	**1.51**
24	*K. intermedius*	AAB	B.UL	GYC	**1.86**
25	No identified	AAB	B.UL	GYC	**1.58**
26	*K. intermedius*	AAB	B.UL	GYC	**1.87**
27	*L. fusiformis*	Other bacteria	FW.EL	GYC	**2.25**
28	No identified	AAB	FW.UL	GYC	**1.62**
29	No identified	AAB	FW.UL	GYC	**1.54**
30	*K. intermedius*	AAB	FW.UL	GYC	**1.72**
31	*L. fusiformis*	Other bacteria	FW.UL	GYC	**2.22**

AW, alcohol wine; B, beer; FW, fine wine; EL, end of loading phase (FL, fast loading; DL, discontinuous loading); UL, just before unloading. AAB, acetic acid bacteria.

Score values represent the confidence degree for the identification of each isolate. High-confidence (2.00–3.00, green color); medium-confidence (1.99–1.70, yellow color); low-confidence or no organism identification possible (0.00–1.69, red color).

## Discussion

The well-known 16S rRNA amplicon sequencing and MALDI-TOF MS techniques have allowed for comparing the microbial composition of three acetification profiles avoiding the risk of ignoring some unculturable or hardly culturable microorganisms under controlled laboratory conditions, as other author have previously reported in vinegar making (Fernández-Pérez et al., 2010; Vegas et al., 2010; Kim et al., 2013; Trček et al., 2016; Peters et al., 2017; Imchen et al., 2019; Rizo et al., 2020). In this work, these aspects are mainly based on the use of three raw materials as acetification substrates with different nutritional features which may influence the microbiota composition and diversity.

The qualitative and biodiversity analyses yielded some evidence based on the number and distribution of OTUs which might clarify fundamental aspects of the characterization of these raw materials and operating conditions (Figures 4, 5). Although significant numbers of OTUs common to all samples of each acetification process were shown, also high numbers of specific OTUs were found at the end of the loading, especially in beer vinegar. Under our working conditions in which a semi-continuous submerged fermentation is performed, the loading phase refills the tank with fresh medium coming from each raw material. At the end of this phase, the moment of highest ethanol concentration and lowest acidity level of the cycle is reached (García-García et al., 2019; Jiménez-Hornero et al., 2020; Román-Camacho et al., 2022). In this milder environment, this point may be characterized by a higher diversity of microorganisms and, consequently, unique OTUs. Conversely, at the final period of the acetification, both the highest acetification rates and final acidity levels are achieved thus favoring the growth of, exclusively, the best-adapted microbiota (Wang et al., 2015; Andrés-Barrao et al., 2016; Zhu et al., 2018). This event might explain the considerable number of OTUs at the final stage of the process for FW vinegar, a highly efficient acetification substrate (Román-Camacho et al., 2022). The higher number of OTUs could be associated with higher diversity levels in the case of AW and FW vinegar, whereas the opposite occurred for B vinegar: the biodiversity study revealed a lower diversity level at B.EL and an increase at B.UL. Sudden changes caused by the addition of fresh medium might establish a specific dominant microbiota that exploit the nutrients provided particularly by the beer medium, such as fermentable sugars and amino acids (Román-Camacho et al., 2022). Other components such as those containing hops have demonstrated a high antimicrobial ability against the growth of several bacteria and molds, which might promote lower diversity when the beer medium is added (Rodhouse and Carbonero, 2019; Tronina et al., 2020). So, the particular features associated with each raw material apparently influence the number and distribution of OTUs in the vinegar metagenome.

On the other hand, the inoculum exhibited the highest number and biodiversity of OTUs according to the alpha and beta diversity analyses performed, far above the vinegar-making samples (Table 1 and Figure 5). It is worth noting that the original starter culture came from a previous acetification process for making wine vinegar which was first used for starting the alcohol wine acetification, which subsequently served as a starter for the acetification of natural raw materials (Román-Camacho et al., 2020, 2022). Under this working system, the microbiota comprising the starter culture are subjected to diverse environmental changes due to the switching to different media. This situation might explain the high biodiversity in the starting inoculum and its progressive loss in vinegar-making samples which would vary depending on the raw material. Peng et al. (2015) determined a decrease in the alpha diversity throughout acetic acid fermentation of Chinese vinegar produced by solid-state fermentation and using 454 pyrosequencing.

In order to carry out a more complete taxonomic study that will facilitate a more accurate identification of the vinegar microbiota, 16S rRNA amplicon sequencing and MALDI-TOF MS were applied. The analysis revealed that Proteobacteria was the most frequent taxon including the acetic acid bacteria (AAB) genera *Acetobacter* and *Komagataeibacter* (Figures 6, 7). The latter was the most predominant genus of the metagenome, far above all other taxa in- or outside of the Proteobacteria. Indeed, the high number of *Komagataeibacter* OTUs (431) found among the sequence reads could lead to a higher diversity of the present species within the genus (Supplementary Table 2). Several studies have demonstrated the suitability of some *Komagataeibacter* species for adaptation and survival throughout industrial acetification due to their physiological and growth characteristics, including high ethanol preference, high acetic acid-producing capability, withstanding both low [7–9% (w/v)] and high [10–20% (w/v)] acidity levels, and a constant oxygen requirement (Yamada et al., 2012; Gullo et al., 2014; Qiu et al., 2021; He et al., 2022). Metagenomics studies applied to the elaboration of vinegar by traditional surface culture (Valera et al., 2015; Peters et al., 2017; Milanović et al., 2018) and industrial submerged culture (Trček et al., 2016) have highlighted the presence and function of *Komagataeibacter*, particularly species such as *K. europaeus*, *K. intermedius*, *K. hansenii*, *K. nataicola*, *K. rhaeticus*, and *K. xylinus*. Furthermore, *Komagataeibacter* has been reported in other omics approaches such as transcriptomics (Wang et al., 2021) and metaproteomics (Andrés-Barrao et al., 2016), including our previous works in which the genus accounted for around 90% of total proteins allowing to confirm these results (Román-Camacho et al., 2020, 2021, 2022). Despite the well-known difficulty to recover AAB on solid culture media from industrial bioreactors (Mamlouk and Gullo, 2013; Vegas et al., 2013), MALDI-TOF MS protein fingerprinting succeeded in identifying 7 isolates as the AAB species *K. intermedius*, from FW and B vinegars. This milestone was also achieved by Andrés-Barrao et al. (2013) in industrial vinegar production.

*Acetobacter* was the other AAB genus identified by amplicon sequencing, one of the most prominent taxa of the non-abundant microbiota, mainly in FW vinegar (Figure 7). In contrast to *Komagataeibacter*, *Acetobacter* may lack either the molecular mechanisms of adaptation to a nutritionally poor medium such as AW or the capability to assimilate the excess sugars from the B medium (Román-Camacho et al., 2022). Despite this, the results of differential abundance analysis suggested that *Acetobacter* exhibited a strong ability to survive throughout the process (Figure 8). Most molecular studies have demonstrated that *Acetobacter* species are normally damaged at acidity levels higher than 8–10% (w/v), so they are usually found in wine, cereal, and balsamic traditional vinegars and early stages of those produced by submerged culture, with *A. pasteurianus* being the most reported one due to its notable production of and high resistance to acetic acid (Gullo and Giudici, 2008; Andrés-Barrao et al., 2011, 2013; Gullo et al., 2014; Zhang et al., 2015; Wu et al., 2017). It is worth noting that similar frequencies for *Acetobacter* to those obtained in this analysis (around 2–3%) were showed in our metaproteomic approaches for the same media (Román-Camacho et al., 2020, 2021, 2022). Despite the use of an optimized medium for AAB, none of the isolates were identified as *Acetobacter* by MALDI-TOF MS, which would indicate that these bacteria may be present in a “viable but non-culturable” (VBNC) state in competition with other microbial groups (De Roos and De Vuyst, 2018; Milanović et al., 2018).

On the other hand, 16S rRNA amplicon sequencing revealed the presence of archaea from the phylum Thaumarchaeota with, more specifically, members of the Nitrososphaeraceae family as the main representatives (Figures 5, 7). To our knowledge, no studies have reported the presence of archaea in industrially produced vinegar, and only Wu et al. (2017) reported by metagenomics “shotgun” sequencing that around 8% of a traditional cereal vinegar microbiota were identified as archaea. Nitrososphaeraceae has been recently found as a part of microbial communities of marine water surfaces and soils by metagenomics and metatranscriptomics (Clark et al., 2021; Jain and Krishnan, 2021). These microorganisms obtain energy by oxidizing ammonia to nitrite aerobically, thereby fixing CO_2_, but growth depends on the addition of small amounts of organic acids (Stieglmeier et al., 2014). Future studies based on functional analyses will be necessary to clarify the physiology and role of these archaea in a medium such as vinegar, both in the raw material and throughout the acetification process.

Many other groups of bacteria, different from AAB, were also identified by 16S rRNA amplicon sequencing. Regarding *Cetobacterium* and *Rhodobacter*, no reports in vinegar have been found for these microorganisms, but because they were present in most of the samples, mainly in those taken along the acetification process, they might play a prominent role in the microbial community. These genera have been associated with the use of diverse strategies for the production and assimilation of acetate, respectively (Erkal et al., 2019; Bhute et al., 2020; Xie et al., 2022). Conversely, the presence of *Bacillus* (e.g., *Bacillus amyloliquefaciens*) has been previously reported in vinegar, concretely, Zhang et al. (2017) showed that they may contribute to the production of acetoin and tetramethylpyrazine, thus enhancing the organoleptic properties, mainly the flavor, of final Chinese vinegars. Moreover, a high concurrence of *Bacillus* was found in biofilms of strawberry vinegar (Valera et al., 2015) and seed vinegar (Milanović et al., 2018). *Sphingomonas* were reported in vinegar for the first time in surface vinegar and, due to the ability of some species to produce extracellular polysaccharides, its presence was attributed to the contribution to the vinegar biofilm formation (Huang et al., 2016; Milanović et al., 2018). To our knowledge, this is the first time that these bacterial genera are identified in submerged culture vinegar, however, further assays could facilitate a better understanding of the strategies used for minor fractions of these microorganisms to survive throughout acetification.

In addition to AAB, other microorganisms were identified both by 16S rRNA amplicon sequencing and MALDI-TOF MS and, particularly, by the latter method (Table 2). These isolates belonged mainly to an endophyte bacteria named *L. fusiformis*, which was widely reported in AW vinegar and some samples of FW vinegar. Although this microorganism has not been well-studied, some works have detailed its role in the production of acetic acid for enhancing signaling pathways to respond to different stressors in plants (Goud et al., 2014; Zhu et al., 2021). Furthermore, one single OTU has been associated with the *Lysinibacillus* genus through 16S rRNA amplicon sequencing (Supplementary Table 2). Despite the low frequency displayed in metagenomics, this microorganism may present appropriate growing conditions for the development in solid media, although its role in the submerged vinegar production requires further studies.

## Conclusion

A strategy applying two different “omics” tools has been carried out to identify the present microbiota during the submerged acetification process of three different raw materials, a synthetic medium and two natural substrates. Metagenomics by 16S rRNA amplicon sequencing and protein fingerprinting by MALDI-TOF MS were implemented, leading to novel results about the microbial composition of vinegar. Metagenomics revealed that along the time courses of the three acetification processes studied, the vinegar microbiota was mainly composed of the AAB genus *Komagataeibacter*, which dramatically outnumbered the rest of the taxa, and a minor fraction of microorganisms including the AAB genus *Acetobacter*, many other groups of bacteria, and even microorganisms belonging to the archaea, mainly Nitrososphaeraceae. Regarding AAB groups, these results allowed us to confirm the composition of the predominant microbiota obtained in our previous metaproteomics approaches. MALDI-TOF MS also confirmed the dominance of *Komagataeibacter* allowing the identification of a specific species, *K. intermedius*. 16S rRNA amplicon sequencing has allowed the detection of a larger number of taxonomic groups, up to genus level, than most DNA-based methods used during the production of vinegar. MALDI-TOF MS may be an easy, cheap, and quick method to complement the identification analysis, allowing to reach the species or strain level. However, the cultivability of the microorganisms in solid media as well as comprehensive databases to compare the peptide mass fingerprints are necessary. The combination of a culture-independent technique and a culture-dependent method, as done in the present work, allows the generation of a broader picture of the industrial production of vinegar. The finding of, not only the main AAB group responsible for the acetification but also of new microbial groups, such as other bacteria and archaea, which were not reported to date in industrial vinegar produced by submerged culture, encourages to perform further studies to understand their role in the microbial community. In addition, new insights on the characterization of the raw materials used have been obtained. This combined “omics” approach may have a biotechnological potential for the vinegar-making industry and even in other related food microbiology fields.

## Data availability statement

The data presented in this study are deposited in the Sequence Read Archive (SRA) repository (NCBI), accession number: PRJNA891065 (https://www.ncbi.nlm.nih.gov/sra/).

## Author contributions

JR-C: methodology, validation, formal analysis, data curation, writing—original draft preparation, and visualization. IG-G and JM: conceptualization, investigation, resources, writing—review and editing, supervision, project administration, and funding acquisition. IS-D: methodology, validation, formal analysis, conceptualization, and visualization. AE and WL: validation and supervision. TG-M: conceptualization, data curation, validation, and supervision. All authors contributed to the article and approved the submitted version.

## Footnotes

1
https://qiime2.org/

2
https://github.com/benjjneb/dada2
