# Supplementary material for: Combining omics tools for the characterization of the microbiota of diverse vinegars obtained by submerged culture: 16S rRNA amplicon sequencing and MALDI-TOF MS
Source: Front Microbiol. 2022 Dec 7;13:1055010. doi: 10.3389/fmicb.2022.1055010 (PMC9767973; doi:10.3389/fmicb.2022.1055010)
Supplement: Supplementary file 1 [file Table_1.DOCX]

**Supplementary Table 1**. Reads obtained after Illumina amplicon sequencing and amplicon sequence variants (ASVs) finally resulting from filtering, denoising, merging, and chimera filtering procedures. AW, alcohol wine; B, beer; FW, fine wine; EL, end of loading phase (FL, fast loading; DL, discontinuous loading); UL, just before unloading.

|  |  |  |  |  |  |
| --- | --- | --- | --- | --- | --- |
| Sample | 1. Reads | 2. Filtering | 3. Denoising | 4. Merging | 5. Chimera filtering |
| Inoculum.1 | 184261 | 166841 (90.6%) | 162560 (88.2%) | 156532 (85.0%) | 154529 (83.9%) |
| Inoculum.2 | 219463 | 209788 (95.6%) | 204428 (93.1%) | 193516 (88.2%) | 187454 (85.4%) |
| Inoculum.3 | 211731 | 202638 (95.7%) | 197197 (93.1%) | 186220 (88.0%) | 179895 (85.0%) |
| AW.FL.1 | 214228 | 203914 (95.2%) | 203046 (94.8%) | 201998 (94.3%) | 201154 (93.9%) |
| AW.FL.2 | 203070 | 192908 (95.0%) | 191944 (94.5%) | 190790 (94.0%) | 189289 (93.2%) |
| AW.FL.3 | 209379 | 199169 (95.1%) | 197615 (94.4%) | 195538 (93.4%) | 192976 (92.2%) |
| AW.FL.4 | 204656 | 194625 (95.1%) | 193092 (94.3%) | 190950 (93.3%) | 188256 (92.0%) |
| AW.DL.1 | 212847 | 202637 (95.2%) | 201873 (94.8%) | 200811 (94.4%) | 198771 (93.4%) |
| AW.DL.2 | 211649 | 201729 (95.3%) | 200929 (94.9%) | 200123 (94.6%) | 199466 (94.2%) |
| AW.DL.3 | 205109 | 194764 (95.0%) | 193828 (94.5%) | 191927 (93.6%) | 190002 (92.6%) |
| AW.UL.1 | 206885 | 197304 (95.4%) | 196480 (95.0%) | 195250 (94.4%) | 193867 (93.7%) |
| AW.UL.2 | 208403 | 198466 (95.2%) | 197177 (94.6%) | 195569 (93.8%) | 194353 (93.3%) |
| AW.UL.3 | 219790 | 209353 (95.3%) | 207960 (94.6%) | 206275 (93.9%) | 204711 (93.1%) |
| B.EL.1 | 213290 | 203004 (95.2%) | 202011 (94.7%) | 200351 (93.9%) | 198187 (92.9%) |
| B.EL.2 | 202849 | 193643 (95.5%) | 192608 (95.0%) | 190958 (94.1%) | 189512 (93.4%) |
| B.EL.3 | 208086 | 198927 (95.6%) | 197997 (95.2%) | 196818 (94.6%) | 196010 (94.2%) |
| B.EL.4 | 208316 | 199323 (95.7%) | 198484 (95.3%) | 197663 (94.9%) | 196811 (94.5%) |
| B.UL.1 | 213583 | 203773 (95.4%) | 202409 (94.8%) | 200085 (93.7%) | 198184 (92.8%) |
| B.UL.2 | 214472 | 204834 (95.5%) | 202152 (94.3%) | 197470 (92.1%) | 194760 (90.8%) |
| B.UL.3 | 207689 | 197863 (95.3%) | 195455 (94.1%) | 191558 (92.2%) | 188744 (90.9%) |
| FW.EL.1 | 218750 | 208264 (95.2%) | 206276 (94.3%) | 202816 (92.7%) | 197451 (90.3%) |
| FW.EL.2 | 218473 | 208391 (95.4%) | 206949 (94.7%) | 205107 (93.9%) | 200021 (91.6%) |
| FW.EL.3 | 211723 | 201522 (95.2%) | 200549 (94.7%) | 199237 (94.1%) | 194768 (92.0%) |
| FW.UL.1 | 218671 | 207879 (95.1%) | 206088 (94.2%) | 202789 (92.7%) | 198847 (90.9%) |
| FW.UL.2 | 207785 | 198366 (95.5%) | 197352 (95.0%) | 196081 (94.4%) | 193134 (93.0%) |
| FW.UL.3 | 217756 | 207336 (95.2%) | 206397 (94.8%) | 204894 (94.1%) | 202957 (93.2%) |
|  |  |  |  |  |  |
